# Artificial Intelligence in Sports Medicine: Reshaping Electrocardiogram Analysis for Athlete Safety—A Narrative Review

**DOI:** 10.3390/sports12060144

**Published:** 2024-05-26

**Authors:** Alina Maria Smaranda, Teodora Simina Drăgoiu, Adela Caramoci, Adelina Ana Afetelor, Anca Mirela Ionescu, Ioana Anca Bădărău

**Affiliations:** 1 Discipline of Sports Medicine, Carol Davila University of Medicine and Pharmacy, 050474 Bucharest, Romania; adela.caramoci@umfcd.ro (A.C.); anca.ionescu@umfcd.ro (A.M.I.); 2 Sports Medicine Resident Doctor, Carol Davila University of Medicine and Pharmacy, 050474 Bucharest, Romania; teodora_simina_ionescu@yahoo.com; 3 National Institute of Sports Medicine, 022103 Bucharest, Romania; 4 Department of Thoracic Surgery, “Marius Nasta” National Institute of Pneumology, 050159 Bucharest, Romania; afetelorana@gmail.com; 5 Department of Physiology, Carol Davila University of Medicine and Pharmacy, 050474 Bucharest, Romania; anca.badarau@umfcd.ro

**Keywords:** artificial intelligence, pre-participation examination, athlete’s heart, sudden cardiac death, sports cardiology

## Abstract

Artificial Intelligence (AI) is redefining electrocardiogram (ECG) analysis in pre-participation examination (PPE) of athletes, enhancing the detection and monitoring of cardiovascular health. Cardiovascular concerns, including sudden cardiac death, pose significant risks during sports activities. Traditional ECG, essential yet limited, often fails to distinguish between benign cardiac adaptations and serious conditions. This narrative review investigates the application of machine learning (ML) and deep learning (DL) in ECG interpretation, aiming to improve the detection of arrhythmias, channelopathies, and hypertrophic cardiomyopathies. A literature review over the past decade, sourcing from PubMed and Google Scholar, highlights the growing adoption of AI in sports medicine for its precision and predictive capabilities. AI algorithms excel at identifying complex cardiac patterns, potentially overlooked by traditional methods, and are increasingly integrated into wearable technologies for continuous monitoring. Overall, by offering a comprehensive overview of current innovations and outlining future advancements, this review supports sports medicine professionals in merging traditional screening methods with state-of-the-art AI technologies. This approach aims to enhance diagnostic accuracy and efficiency in athlete care, promoting early detection and more effective monitoring through AI-enhanced ECG analysis within athlete PPEs.

## 1. Introduction

Cardiovascular health is a critical concern for sports medicine physicians and a key component of athletic performance. Sudden cardiac death (SCD) is a leading cause of mortality among athletes and may result from congenital heart diseases such as hypertrophic cardiomyopathy (HCM), channelopathies, and arrhythmogenic right ventricular cardiomyopathy [1]. Sports medicine doctors aim to prevent SCD through regular and effective screening. The pre-participation examination (PPE), which includes a structured protocol proposed by the International Olympic Committee (IOC) and the European Federation of Sports Medicine Associations (EFSMA), prioritizes the screening for cardiovascular diseases [2]. Sports and Exercise Medicine (SEM) professionals play a critical role in this process, ensuring athletes’ safety through systematic health screenings that encompass cardiovascular assessments including medical history and a resting 12-lead electrocardiogram (ECG), occasionally supplemented by additional investigations. ECG, a non-invasive, inexpensive, and easily performed diagnostic tool, can provide critical information about a patient’s cardiovascular health and uncover potentially dangerous cardiac diseases. A study assessing the effectiveness of pre-participation screening algorithms highlights the limitations of questionnaire-based approaches, showing that cardiovascular abnormalities could be missed without thorough medical evaluation, underscoring the importance of comprehensive PPE [3]. Specialized protocols for interpreting athletes’ ECGs have been established [1]. Athletes’ ECGs often exhibit characteristics indicative of cardiac adaptation to increased physical demands, which, while potentially pathological in the general population, are normal and harmless for highly trained athletes. Consequently, the European Society of Cardiology (ESC) has proposed a standard for ECG interpretation in athletes [1]. Cardiac adaptation is beneficial for physical performance; however, subtle deviations in a normal ECG may indicate serious cardiac conditions, making it imperative for physicians to identify them accurately. The challenge lies in differentiating between normal cardiac adaptations in athletes and abnormalities that suggest potential disorders, requiring a comprehensive understanding of both the physiological changes in an athlete’s heart and the indicators of possible medical conditions, thereby necessitating a detailed step-by-step multimodality approach for accurate diagnosis [2,4,5].

The field of artificial intelligence (AI) is rapidly expanding, and its applications are being explored in various sectors of the healthcare system [6]. Many countries are grappling with immense workloads in their medical systems, frequently compounded by staffing shortages. In this context, AI has the potential to alleviate this burden and enhance the efficiency of medical care. The use of AI in interpreting ECGs has a considerable history [7] and recent advancements highlighted by Adasuriya and Haldar [8] demonstrate AI’s potential to significantly improve the detection and analysis of complex cardiac conditions through advanced algorithms, offering a more sophisticated, precise, and predictive approach to ECG interpretation in athletes [8].

The leading cause of mortality in athletes during sports and exercise is cardiovascular-related sudden death [1,9,10,11], emphasizing the critical need for effective screening methods. We aimed to explore the utility of AI in enhancing electrocardiogram ECG screenings during PPE for athletes. By integrating AI technologies, we aim to advance the early detection and prevention of cardiac anomalies, thereby significantly improving the safety and well-being of athletes engaged in intense physical activities. The potential of AI to augment the diagnostic accuracy and efficiency of ECG interpretations offers a promising avenue to transform traditional screening practices into more sophisticated, precise, and predictive healthcare interventions. This narrative review will consolidate current research and delve into the implications of AI-enhanced ECG screening within sports medicine, highlighting its benefits not only for the general population but also specifically for the athletic community. By providing a comprehensive overview of current innovations and identifying future advancements, this study supports SEM professionals in bridging the gap between conventional methods and cutting-edge technologies.

## 2. Methods

We reviewed articles published within the past 10 years, utilizing the PubMed and Google Scholar databases. The search was performed using the following keywords: “artificial intelligence” and “ECG”, “machine learning” and “ECG”, “deep learning” and “ECG”, “artificial intelligence” and “sports cardiology”, “machine learning” and “sports cardiology”, and “deep learning” and “sports cardiology”. After consulting MeSH, we found an exhaustive list of entry terms. However, we deemed a broader search too extensive and not sufficiently focused on our research questions. We included only articles written in English, comprising original studies and reviews. We selected studies in alignment with the ESC standards for ECG interpretation in athletes, particularly focusing on research that addresses borderline and abnormal ECG findings to ensure clinical relevance and applicability to sports cardiology. The research question was structured as recommended by formulating the population to comprise patients undergoing ECG testing for cardiovascular diseases. The intervention involved utilizing AI technology for ECG interpretation, while the control encompassed traditional ECG interpretation by professionals.

## 3. Results

The complexity of AI technologies involved in ECG interpretation is not the focus of this article. However, acknowledging that multiple mechanisms enable this process, each with its own limitations and advantages, is important. Table 1 summarizes the key studies reviewed, presenting their design, main findings, utilized AI technologies, and their applicability and limitations in sports cardiology. Subsequent sections provide an in-depth discussion of how AI is transforming ECG interpretation. This analysis addresses the technological advancements, challenges faced, and future prospects as suggested by the literature. It critically examines the role of AI in sports medicine, focusing on its practical implementation to enhance athlete safety and performance in clinical settings.

AI comprises technologies that process data and can emulate human cognitive processes [12]. This term is often confused with machine learning (ML), a distinct field. ML recognizes patterns from previous inputs, and its outcome generation does not adhere to specific rules. Consequently, ML relies on opaque black-box models that can be challenging to comprehend, making them less trustworthy for both doctors and patients [12].

Moreover, ML is prone to biases if the input data are not representative of the general population or do not reflect local management protocols. This issue is particularly biased in the context of rare diseases or underrepresented groups. ML encompasses both supervised and unsupervised learning systems [12]. Deep learning (DL), a subset of ML, simulates human cognitive processes through artificial neural networks (ANNs) with intricate mechanisms that produce outcomes [12,13]. DL does not require large, labeled datasets to process raw data and infer conclusions [13]. In contrast to traditional models, DL learns to internalize data representation, eliminating the need for human-selected features. This approach is highly effective in ECG analysis, enabling the detection of complex patterns without human bias [14].

Convolutional neural networks (CNNs), which optimize both data representation and analytical rules [14], are frequently employed for this purpose. DL is used in ECG analysis for screening a variety of cardiac and non-cardiac conditions. The selection among DL, CNN, and recurrent neural networks depends on the available datasets [13]. Widely used datasets include PhysioNet, the MIT-BIH arrhythmia database, the MIT-BIH AF database, the MIMIC database, and Computing in Cardiology [13,15]. CNNs can also serve as a DL technique [15]. Unsupervised learning, another promising concept in cardiology, can identify latent structures and relationships in datasets without the need for labeled observations. This method has the potential to transform cardiology by enabling “precision phenotyping”, paving the way for precision medicine [9].

Common parameters used to assess the efficacy of AI methods in ECG include precision or positive predictive value, sensitivity, specificity, area under the curve (AUC), c-statistics, and F1-statistics [13]. 

Various AI technologies have been developed to identify different cardiac diseases using ECG. Bellfield et al. [17] highlighted a critical challenge in ML applications for sports cardiology: small sample sizes and imbalanced data often lead to overfitting and reduced generalizability, undermining the reliability of models for accurately diagnosing heart defects in athletes. The extensive analysis comprised 28 research studies. The review revealed a significant trend in sports medicine, particularly regarding the understanding of the athlete’s heart, with a pronounced shift towards adopting ML techniques. This trend was evidenced by 57% of the studies, characterized by their use of ML for developing models to address specific cardiac health-related challenges or for evaluating ML implementation in research areas with similar focuses [17].

Notably, Barbieri et al. [18] conducted an extensive analysis involving 26,002 participants to evaluate cardiovascular risk among athletes. Their methodology incorporated the analysis of tabular records and ECG characteristics, applying both decision tree and logistic regression models. The logistic regression model demonstrated notable efficacy with an AUC of 0.78. By contrast [17,18], Castillo-Atoche et al. [19] employed a more substantial dataset, consisting of 56,542 ECG samples from 487 patients. They utilized a CNN for real-time arrhythmia prediction. Their findings revealed an impressive accuracy of 94.3% on the training set and 93.9% on the test set, with these datasets originating from wearable devices [17,19].

The role of AI in ECG interpretation is to redefine diagnostic approaches, particularly through its ability to identify both known and novel physiological patterns, thereby enhancing diagnostic accuracy [39]. Key to this evolution are physiological experimentation and in silico modeling tools, such as saliency maps and generalized adversarial networks, which are crucial for refining AI-enhanced ECG (AI-ECG) models [39]. 

### 3.1. Cardiac Structural and Electrical Alterations

#### 3.1.1. Atrial Fibrillation

Atrial fibrillation (AF) is the most commonly encountered arrhythmia [40], with a significant portion of studies focusing on the ability of AI-ECG to monitor this condition. Research demonstrates AI-ECG’s effectiveness in detecting AF, including concealed paroxysmal AF, which does not appear on an ECG during examination [7,12,14,15,20,21,22,40,41]. The early diagnosis of AF through AI-ECG could decrease the incidence of unexplained embolic strokes and overall mortality associated with AF [14,41]. AI-ECG may also serve as a risk stratification tool for patients with AF, with or without additional clinical data, aiding clinicians in determining the necessity for anticoagulation therapy [7,20,40,41]. While some researchers argue that AI-ECG can identify “silent” AF but cannot predict its onset [14], others have found that DL algorithms can predict both the risk of AF occurrence and the progression from transient to persistent AF [7]. The most frequently cited AI-ECG method for AF identification and stroke risk assessment employs CNN techniques [13,14,21]. CNN is generally the preferred AI method for ECG analysis [13]. Attia et al. [21] conducted a retrospective study that demonstrated an AI-ECG technique’s ability to detect AF with an accuracy of 79.4% and an AUC of 0.79 [21]. AI techniques in wearable devices, such as smartwatches, have received FDA approval for AF detection using a single-lead ECG mechanism [15]. A study utilizing a CNN-trained single-lead recorder system with the AliveCor dataset screened for AF, showing AI techniques’ promising accuracy compared to insertable cardiac monitor devices [13]. A prospective study indicated a fivefold increase in the risk of AF diagnosis during 30-day continuous ambulatory ECG monitoring for individuals identified as high-risk by a normal, single AI-ECG scan [12]. 

#### 3.1.2. Channelopathies

In children and adolescents, SCD is frequently caused by channelopathies such as long QT syndrome (LQTS) and Brugada syndrome (BrS) [42]. The risk of SCD is elevated in young athletes because intense physical activity may trigger ventricular arrhythmias associated with these conditions. The application of AI-ECG is vital for the accurate detection of these cardiac abnormalities. AI-ECG has been demonstrated in some retrospective studies to detect long QT intervals in patients without measurements above the upper limit on the ECG trace by analyzing the T-wave [7,41].

Certain medications, such as azithromycin and hydroxychloroquine, which were widely used during the COVID-19 pandemic, as well as antiarrhythmic drugs, can prolong the QT interval. A CNN-based AI study that analyzed the ECGs of over 2000 patients concluded that AI-ECG significantly improved the diagnosis of concealed LQTS compared to traditional methods. AI-ECG was particularly effective in identifying LQTS in patients with normal QT intervals and distinguishing between different LQTS subtypes [23].

A comprehensive review of studies highlighted the significant potential of AI, particularly DL and neural networks, in diagnosing and monitoring LQTS [24]. The integration of AI into clinical practice promises to enhance the precision of LQTS diagnosis, improve patient outcomes, and potentially reshape the approach to cardiac arrhythmia monitoring and treatment [24]. Therefore, the capability of AI-ECG to predict drug concentrations and prevent side effects [14]. Long QT syndrome or short QT syndrome can cause life-threatening ventricular arrhythmias, which may be triggered by certain sports, including swimming. Evidence suggests that AI technology, integrated into mobile applications, can be used to detect abnormal QT interval prolongation [12].

The BrAID project highlights the capabilities of echo state networks, a type of recurrent neural network, in diagnosing type 1 BrS through ECG pattern analysis. Employing ECGs from diverse patient cohorts, the echo state network model exhibited robust accuracy in identifying type 1 BrS, yielding comparable outcomes when analyzing either three leads (79.21%) or solely the V2 lead (80.20%) [25].

Analyzing over 2000 ECGs from 157 patients, Nakamura et al.’s study [26] demonstrated the potential of an AI-enabled algorithm, specifically a CNN, in predicting ventricular fibrillation in patients with BrS. The model’s high precision in predicting the presence of ventricular fibrillation is particularly noteworthy, offering a promising tool for the early detection and prevention of SCD in patients with BrS [26].

Early detection and management of BrS are crucial in the ECG screening of athletes. Many cases of BrS remain undiagnosed due to the absence of ECG changes. However, the study by Melo et al. [27] yielded promising results by employing a deep neural network model to detect BrS signatures in standard ECGs, eliminating the need for proarrhythmic drug challenges. This model successfully identifies subtle ECG abnormalities that are often undetected by physicians [27].

Zanchi et al. [28] provided an interesting perspective in their study that employed an AI-based ML model to diagnose BrS by analyzing P-wave characteristics in ECGs. The study’s successful use of AI to identify BrS solely based on P-wave attributes represents a significant advancement in non-invasive, AI-guided BrS diagnosis [28].

Numerous review articles have cited studies demonstrating the potential of AI-ECG to recognize non-cardiac variables, such as serum potassium levels, particularly hyperkalemia, using CNN/DL [7,12,13,14,41], as well as age, sex [13,41], and glycemia [12,15]. Furthermore, Kwon et al. [29] developed a DL model that uses ECGs to detect and monitor electrolyte imbalances, showing high accuracy in both internal and external validations across various electrolyte conditions, potentially offering a reliable, non-invasive tool for daily clinical use [29]. Detecting electrolyte imbalances through ECG adds significant value to sports medicine. It has great potential for monitoring athletes experiencing electrolyte imbalances due to strenuous exercise or the misuse of performance-enhancing substances. Additionally, it proves useful in identifying reversible causes of long QT and Brugada-like patterns [1], thus avoiding costly and unnecessary further evaluations and ensuring efficient, accurate diagnoses in sports medicine.

#### 3.1.3. Hypertrophic Cardiomyopathy

In athletes, isolated QRS voltage criteria indicating left ventricular hypertrophy usually do not signify pathology, as they are rarely associated with HCM. Pathological left ventricular hypertrophy, on the other hand, often presents with additional ECG abnormalities such as T-wave inversion, ST-segment depression, and pathological Q waves. High QRS voltages alone, without other suggestive modifications, are typically considered normal adaptations in athletes [1]. 

HCM is a congenital heart disease that can be difficult to distinguish from athletic left ventricular hypertrophy, which results from increased physical activity. AI-ECG was proven to be a useful method to distinguish HCM from cardiac adaptation [14,21]. DL can detect this condition and other cardiomyopathies [13]. Studies also demonstrated the utility of the CNN modality, combined DL and ML methods, or modified CNN architectures in identifying HCM [15]. Recent research indicated that AI-ECG algorithms are more accurate in detecting HCM in young adolescents and adults but less effective in small children, due to the complex ECG characteristics present in small children that AI mechanisms do not take into account [30]. 

The study by Siontis et al. [31] has significant implications for ECG screening in athletes. Diagnosing HCM in this population can be challenging due to their unique cardiac adaptations. The application of AI, specifically CNN models, to interpret ECGs represents an innovative approach to enhance screening accuracy. The study demonstrated that the one-lead, median-beat CNN model achieved a high accuracy level, with an AUC of 0.90, in detecting HCM, particularly when analyzed through saliency maps. This model can effectively identify HCM by focusing on specific ECG segments, such as the ST-T segment, atrial depolarization, and the QRS complex. The main outcome indicates that ventricular repolarization is the primary region of interest for HCM detection [31]. 

Adetiba et al. [32] developed an automated model to detect heart defects in athletes using ECG and ANN. The study included 40 participants, both athletes and non-athletes, to encompass various heart conditions such as tachyarrhythmia, bradyarrhythmia, and HCM. The ECG data were pre-processed and analyzed using ANNs, with the Levenberg–Marquardt algorithm demonstrating superior performance. The study successfully developed a neural network model achieving an accuracy, sensitivity, and specificity of 90.00%, 91.96%, and 97.06%, respectively [32]. 

Lyon et al. [33] employed ML to identify four distinct HCM phenotypes using QRS morphology and T-wave biomarkers from high-fidelity ECGs. Their findings showed variations in HCM risk-SCD scores and left ventricular hypertrophy distributions. Group 1A, characterized by normal QRS and primary T-wave inversion, displayed the highest risk, with a combination of septal and apical hypertrophy [33]. This study highlights the potential of ML and the relevance of ECG phenotyping in sports cardiology to detect subtle cardiac abnormalities in athletes, which is vital for early intervention.

In the comprehensive study by Koo et al. [34], involving a cohort of 3060 patients diagnosed with HCM and 63,941 control individuals, the authors rigorously tested a model that demonstrated high effectiveness, with an AUC of 0.96, a sensitivity of 87%, and a specificity of 91%. The model performed exceptionally well in younger patients, achieving 95% sensitivity and 92% specificity [34]. Although further refinement and external validation are required, this model, tested on a significantly larger population than is typical in such studies, shows considerable promise for HCM screening applications.

#### 3.1.4. Valvular Disease

AI-ECG is efficient in detecting valvular diseases from early, asymptomatic stages, and its effectiveness has been confirmed for the most common types of valvulopathies, namely, aortic stenosis and mitral regurgitation [41]. Other studies suggested that both 12-lead and single-lead AI-ECG can predict the presence of aortic stenosis or mitral regurgitation when these conditions are severe [7]. DL techniques have been tested for identifying valvulopathies with promising results. CNNs have been used to predict the presence of atrial stenosis using ECG data, such as T-wave abnormalities in V1–V4 [13]. CNNs have also been employed to detect mitral regurgitation by analyzing the P-wave flattening pattern and T-wave anomalies in patients with valvulopathy, as well as ORS complex data in those without mitral regurgitation [13].

### 3.2. Role of AI-ECG in Detecting Heart Failure and Arrhythmias

Due to its capability to detect reduced left ventricular ejection fraction and to differentiate between ejection fractions below and above 35%, AI-ECG can be effectively utilized in the emergency department [7,41]. AI-ECG can identify impairment of left ventricular systolic function even in the subclinical initial phase, where echocardiogram measurements may not indicate a reduced fraction [14]. Some false-positive results from AI-ECG were monitored over several years to determine whether the patients developed heart failure; the findings indicated that the patients were at potential risk of developing cardiac failure [14]. 

The theory is that ECG changes in heart failure could be explained by impulse and conduction alterations, as well as atrial and ventricular remodeling. However, it is crucial to avoid confounding factors such as arrhythmias, poor electrode contact, paced rhythms, and incorrect electrode placement [43]. The accuracy of AI-ECG in detecting impaired left ventricular ejection fraction has been demonstrated in both sinus and AF rhythms [30]. Left ventricular systolic dysfunction can be identified by AI algorithms, even in one-lead ECG scans [7]. AI algorithms can predict the need for hospital admission due to heart failure. One of the key advantages of AI-ECG is its ability to detect a low ejection fraction, identifying new cases of heart failure that might be missed if only an ECG were used without additional diagnostic tools [12]. 

Several studies demonstrated that AI-ECG methods surpass doctors from various specialties in accurately detecting arrhythmias. Martinez-Selles et al. reported a 98% accuracy rate in the detection and classification of arrhythmias [7]. AI has proven effective in identifying life-threatening arrhythmias, potentially reducing analysis time in emergency rooms and aiding in pinpointing the origin of ventricular ectopic beats [7]. 

Considering the advancements in AI-ECG technology and its proven accuracy in detecting heart failure, left ventricular dysfunction, and life-threatening arrhythmias, the future incorporation of Internet of Things wearables into sports cardiology holds immense promise. These devices may soon enable us to predict and potentially prevent cardiac arrest during training or matches for athletes. This represents a pinnacle achievement in the integration of AI within sports medicine and athlete safety.

### 3.3. Internet of Things Wearables

Internet of things wearables represent a technological evolution, offering continuous health monitoring and seamless data integration. They provide a convenient method for monitoring various parameters, commonly used to track heart rate, heart rate variability, and R-R intervals [37]. These metrics are instrumental in guiding training sessions, evaluating the body’s response to physical activity, assessing cardiovascular capacity for endurance training, estimating energy expenditure, and controlling training intensity based on heart rate to engage specific energy-providing mechanisms. Such monitoring can be achieved through ANN, support vector regression, or evolutionary neural networks. The adaptive particle swarm optimization dataset can be utilized to develop training models for athletes [37]. 

The Apple Heart Study pioneered digital health research, demonstrating the feasibility and effectiveness of large-scale, remote health monitoring studies. In a study involving 419,297 participants using a smartwatch app to detect irregular pulses, 0.52% received notifications for potential atrial fibrillation. Among those notified of an irregular pulse, the positive predictive value was 0.84 (95% CI, 0.76 to 0.92) for concurrent atrial fibrillation on the ECG with a subsequent irregular pulse notification. This indicates the potential of wearable technology for large-scale, remote monitoring of cardiac health [38]. Such technology could be beneficial in providing real-time data and early detection of cardiac irregularities, thereby enhancing preventive care and optimizing athletic performance.

Several studies highlight the capabilities of AI technologies in wearables, including the detection of premature atrial and ventricular contractions with an accuracy exceeding 97% [15]. However, other papers indicate that wearable devices possess limited capabilities in identifying arrhythmias other than AF [15]. 

An extensive study, which included more than 83,000 participants from two large UK Biobank sub-studies, highlights the significant association between premature atrial contractions and ventricular contractions (PVCs) detected in 15-s, single-lead ECGs and the increased future risk of cardiovascular diseases, including atrial fibrillation and heart failure [35]. 

In athletes, multiple PVCs are rare but may indicate underlying heart disease, particularly when originating from the right ventricular outflow tract with a prolonged QRS. Evaluation should include Holter monitoring, echocardiography, and stress testing. For athletes with high arrhythmic risk profiles, further investigation such as cardiac MRI and electrophysiology studies may be needed. Detraining can be considered in managing athletes with PVCs, as it may help in selected high-risk cases by suggesting a benign nature of the arrhythmia if the burden decreases during the detraining period. For atrial tachyarrhythmias such as sinus tachycardia, SVT, atrial fibrillation, and flutter, assessing for structural heart disease and potential genetic causes is essential. Ventricular arrhythmias, including couplets, triplets, and non-sustained ventricular tachycardia, always require comprehensive investigation due to their link with cardiac pathology and the risk of SCD [1,44,45,46]. 

Therefore, these studies offer a promising perspective for the use of wearable ECG screening in athletes to detect arrhythmias and assess their response to exercise. However, they also raise important considerations regarding the potential anxiety and over-investigation that athletes might experience upon discovering arrhythmic abnormalities on their ECGs.

### 3.4. eSports Athletes

Cardiovascular health in eSports athletes is a crucial concern, as they may have either known or hidden heart conditions [47]. The competitive nature of playing against human opponents typically stimulates the sympathetic nervous system, leading to changes in cardiovascular function [47,48]. The stress and physiological demands of gaming significantly influence ECG patterns in these athletes, often resulting in elevated heart rates and blood pressure during gaming sessions [40]. Furthermore, research conducted by Rossoni et al. suggests that eAthletes might face a high risk of developing cardiac arrhythmias and other cardiovascular conditions due to factors like mental stress, stimulant use, and prolonged sitting. Therefore, similar to traditional athletes, eAthletes may benefit from PPE, including ECG screening, to identify and manage potential heart-related conditions [47,48]. 

Current research, although limited, suggests potential cardiovascular risks, including an increased incidence of arrhythmias and other heart-related issues. This emerging evidence emphasizes the importance of raising awareness and education among healthcare providers and eSports teams regarding these risks. It also highlights the need to establish specific health and wellness guidelines and screening protocols to protect the cardiovascular health of eSports athletes. Integrating wearable ECG monitors and other advanced technologies into eSports training and competition shows considerable promise for improving player performance and health [49]. 

## 4. Best Practice Example

The Mayo Clinic serves as a best practice example in applying AI to healthcare, particularly through its AI-ECG Dashboard [14]. This internal tool enables retrospective analysis of patient ECGs, offering probabilities for conditions such as LV systolic dysfunction, silent AF, and HCM, along with AI-predicted age and sex. Integrated into the electronic medical record, it allows clinicians to quickly access AI analysis results for all available patient ECGs [14]. Such innovations in AI-ECG technology have significant implications for sports cardiology, especially in the pre-participation ECG screening of athletes, where accurate and rapid assessment is crucial.

In a notable collaboration, Who We Play For joined forces with Amazon web services to develop an innovative ML tool specifically designed for pediatric SCD risk detection through ECG screenings [36]. This endeavor began with the digitization and analysis of ECG traces using a DL algorithm, which initially yielded 78% sensitivity and 90% specificity. The model’s precision was further enhanced to over 93% accuracy, surpassing the capabilities of human interpretation, thanks to input from Who We Play For cardiologists integrated via Amazon Augmented AI [36]. This initiative showcases the use of advanced technology in preventing SCD by screening young athletes with ECG, offering a cost-effective and widely applicable method that could save lives.

## 5. Limitations

This study has several limitations. First, it did not constitute a systematic review encompassing all published data on the topic, potentially leading to the omission of important information. Second, relying on other reviews as data sources implies a dependence on the original authors’ interpretations and analyses, potentially perpetuating biases and selection errors. Third, the majority of the articles reviewed did not focus exclusively on athletes, a critical group in sports medicine. This oversight highlights a significant gap in the literature and underscores the need for further research specifically targeting athlete populations. Despite these limitations, the review aims to underscore the transformative potential of AI in sports medicine, particularly in ECG interpretation, and to encourage the targeted research that could ultimately lead to personalized and precise medical protocols for athlete care.

## 6. Conclusions

The application of AI in ECG interpretation aids in distinguishing between physiological adaptations in athletes and pathological conditions, a critical factor in preventing sudden cardiac events. Nonetheless, employing AI in this field presents several challenges. 

Although AI shows promise in detecting known ECG anomalies, its capability to identify new or poorly understood cardiac traits necessitates further exploration and external validation.

Another issue pertains to the legal and ethical aspects of the medical profession, wherein each decision is the responsibility of the doctor, and liability is attributed to the individual. If AI were the cause of a medical error, it could complicate the determination of human fault. 

AI technologies have demonstrated effectiveness in medicine, and implementing these methods can offer numerous advantages. We discussed AI’s potential to identify pathological features that might be missed by the human eye or easily overlooked, even by experienced professionals. Another important aspect is that some pathologies may not exhibit clinical symptoms at the time of examination, resulting in a lack of abnormal ECG findings. 

This review highlights the transformative potential of AI in sports medicine, particularly regarding ECG interpretation for athletes. AI technologies, such as ML, DL, and neural networks, have demonstrated remarkable proficiency in detecting cardiac conditions, including arrhythmias, channelopathies, HCM, and valvular diseases. The integration of AI into sports cardiology is crucial for two main reasons: it enhances diagnostic accuracy and deepens our understanding of athletes’ cardiac dynamics. This approach promises more personalized and effective cardiac health strategies tailored to athletes [17]. The potential extension of AI capabilities to wearable devices opens new avenues for continuous monitoring and risk assessment in athletes during both training and competition.

**Figure 1:**
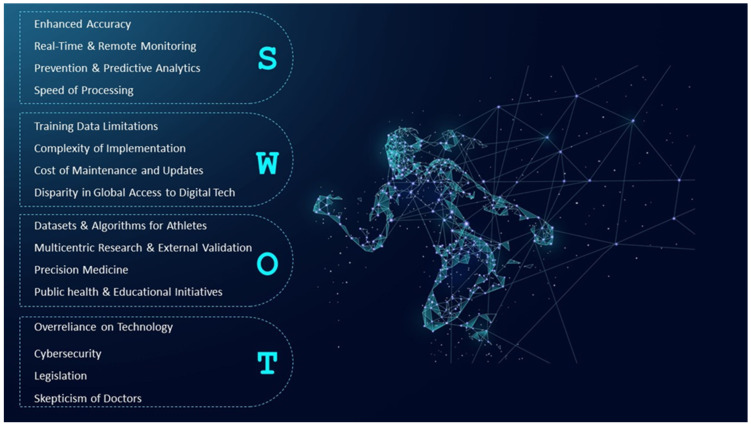
Evaluating the Future of AI in Sports Medicine: A SWOT Perspective.

The SWOT analysis depicted in Figure 1 emphasizes the profound impact of AI on sports cardiology, enhancing cardiovascular monitoring and diagnostics for athletes. It highlights strengths such as improved diagnostic accuracy and real-time data processing capabilities. However, it also points out significant challenges, including substantial costs, data privacy concerns, and potential threats like technological dependency and the risk of data misuse that must be navigated carefully. The opportunities section suggests areas for growth such as personalized athlete models and cross-disciplinary collaborations, enhancing the practical application of AI in sports medicine [50]. This strategic overview supports the aim of the review, which is to integrate AI in ECG screenings during PPE, assisting SEM doctors.

## 7. Directions

Further research is required to demonstrate the cost-effectiveness of implementing AI-based ECG interpretation in athletic populations and to confirm the reliability of this system in the decision-making process. 

AI-driven ECG interpretation algorithms stand out from conventional methods due to their potential for continuous improvement [39]. Once optimized, these models can be adapted for new uses through transfer learning, thereby expanding their applicability, particularly in enhancing the efficacy and accuracy of ECG screening in athletes.

Future AI development should focus on creating customized ECG interpretation algorithms for athletes, incorporating age, sex, race, and sport type. Such a personalized approach will improve the accuracy of detecting cardiac risks, ensuring safer sports participation and more effective monitoring of athletes’ heart health.

## Figures and Tables

**Table 1:** Summary of Key Studies on Cardiovascular Health and Artificial Intelligence Applications in Sports Cardiology.

No	Authors	Study Design	Main Findings	AI Technology Used	Relevance to Sports Medicine	Limitations
1	Danilov A, Aronow WS (2023) [12]	Review	AI in cardiology is at an early stage but has potential benefits in diagnostics and treatments. Key applications include imaging, electrocardiography, wearable devices, risk prediction, and disease classification. Major hurdles remain in model understanding, bias, evaluation, legal and ethical dilemmas.	Machine Learning, Deep Learning, Neural Networks	The insights into AI-driven diagnostics and risk predictions could inform personalized training and rehabilitation programs for athletes, helping prevent cardiovascular issues.	The study notes the lack of extensive clinical trials and the challenges of model interpretability, which could impact the broader adoption and effectiveness of AI technologies in sports medicine.
2	Somani S, et al. (2021) [13]	Review	Reviews the integration of deep learning with electrocardiography, highlighting improved diagnosis and management of cardiac conditions such as arrhythmias, cardiomyopathy, and ischemia. Also notes the importance of large, diverse datasets and the potential of novel clinical applications.	Deep Learning	Can assist in enhancing monitoring and diagnostics in sports settings, particularly for conditions like arrhythmias which may be triggered by intense physical activity.	Challenges include the dependency on large, diverse datasets which may not always be available or of sufficient quality in sports settings, and the need for external validation in sports-specific populations.
3	Konstantinos C. Siontis KC, et al. (2021) [14]	Review	Discusses the transformative effect of AI on ECG interpretation, highlighting its potential to improve diagnostics in cardiovascular medicine. AI techniques can detect patterns in ECGs that are unrecognizable to humans, potentially identifying early signs of diseases like left ventricular dysfunction and hypertrophic cardiomyopathy.	Convolutional Neural Networks	The advanced detection capabilities of AI-enhanced ECGs can be crucial in sports medicine for early identification of athletes at risk of cardiovascular diseases.	As AI-ECG tools continue to evolve, their real-world implementation faces challenges like ensuring data quality, model validation across diverse populations, and integration into clinical workflows.
4	Neri L et al. (2023) [15]	Review	Examines advancements in AI for ECG monitoring via wearable devices, focusing on arrhythmias and coronary artery disease. Highlights the use of deep learning methods such as CNNs and RNNs for improved disease detection and prediction capabilities.	Convolutional Neural Networks (CNNs), Recurrent Neural Networks (RNNs)	Wearable ECG devices with AI can significantly enhance the monitoring and detection capabilities, important for athletes in managing arrhythmias and coronary health.	Limitations of wearable ECGs compared to standard multi-lead ECGs include less data richness and potential inaccuracies in data collection, especially during intense physical activity.
5	Johnson KW, et al. (2018) [16]	Review	Explores AI and ML in cardiology, highlighting how these technologies aid in the management of cardiovascular diseases through improved prediction and personalized treatment plans. Discusses the use of AI in feature selection for models, enhancing predictive accuracy beyond traditional statistical methods, and aiding in the interpretation of complex data from various sources.	Machine Learning, Deep Learning, Neural Networks, Convolutional Neural Networks, Recurrent Neural Networks, Support Vector Machines	Enhances data interpretation and management, crucial for monitoring athletes’ heart health and adapting training programs to individual cardiovascular profiles.	AI reliance on extensive, high-quality datasets, which may not always be available in sports settings, and the general complexity of AI models which require specialized expertise to manage and interpret.
6	Bellfield RAA, et al. (2022) [17]	Review	Reviews the use of ML techniques in researching the athlete’s heart, highlighting the integration of different ML approaches to better understand and manage physiological changes and disease risks due to intense physical training. Discusses current applications, identifies gaps, and suggests future directions for research including the need for larger, diverse datasets and the potential development of models for better disease prediction and management.	Machine Learning, Artificial Neural Networks	Directly addresses the integration of ML in sports cardiology to better understand the physiological impacts of intense training and to improve early diagnostic capabilities.	Highlights the challenges of limited dataset sizes and the need for more comprehensive studies to enhance the validity and applicability of ML models in sports cardiology.
7	Barbieri D, et al. (2020) [18]	Research	Evaluates the impact of resampling techniques on machine learning classification performance in predicting cardiovascular risk in athletes. Demonstrates that techniques like SMOTE can significantly improve sensitivity and predictive accuracy in imbalanced datasets, making it valuable for medical diagnostic systems, particularly in identifying at-risk athletes.	Decision Trees, Logistic Regression, SMOTE	Essential for assessing cardiovascular risk in athletes, enabling early intervention and tailored health management strategies.	While resampling improves classification accuracy, challenges persist in balancing sensitivity and specificity, especially in medical contexts where misclassification costs are high.
8	Caamal-Herrera A, et al. (2022) [19]	Observational Study	Demonstrated an energy-efficient framework for AIoT wearable cardiac arrhythmia detection system. Achieved a high arrhythmia detection precision of 98.6% using convolutional neural networks.	Convolutional Neural Networks	Focuses on real-time ECG monitoring and arrhythmia detection during sports activities, essential for monitoring athletes’ cardiac health.	Study limited to a small cohort of athletes, and did not compare with other diagnostic tools beyond ECG. Potential biases due to small sample size and limited environment test.
10	Noseworthy PA, et al. (2022) [20]	Prospective non-randomized interventional trial	Atrial fibrillation was detected in 1.6% of patients with low risk and 7.6% of patients with high risk. AI-guided screening increased atrial fibrillation detection compared to usual care (10.6% vs. 3.6% in high-risk group; 2.4% vs. 0.9% in low-risk group).	Artificial intelligence algorithm applied to ECG data	Although not directly related to sports medicine, the AI-guided screening approach could potentially be adapted for athletes to screen for cardiac arrhythmias or other heart conditions during routine check-ups or pre-participation evaluations.	Limitations include the potential for false positives or false negatives in AI algorithm predictions, as well as the need for further validation in diverse populations and settings.
11	Martínez-Sellés M, Manuel Marina-Breysse M (2023) [7]	Review	Reviewed the current and future applications of artificial intelligence in electrocardiography, including interpretation and detection of ECG abnormalities, risk prediction, monitoring ECG signals, signal processing, therapy guidance, and integration with other modalities.	Various AI algorithms	Provides insights into the potential use of AI in improving ECG diagnosis and management, which could have implications for sports medicine in detecting cardiovascular abnormalities in athletes.	Does not present original research findings; mainly a review of the existing literature and future perspectives.
12	Attia ZI, et al. (2019) [21]	Retrospective analysis	Developed an AI-enabled electrocardiograph using a convolutional neural network to detect the electrocardiographic signature of atrial fibrillation present during normal sinus rhythm using standard 10-s, 12-lead ECGs.	Convolutional neural network	Could potentially aid in the identification of individuals with atrial fibrillation during normal sinus rhythm, thus providing a rapid, inexpensive means of screening.	Limitations may include the retrospective nature of the analysis, potential biases in the dataset, and the need for further validation in prospective studies and clinical settings.
13	Harmon DM, et al. (2023) [22]	Review	Reviewed specific applications of AI for screening, diagnosis, and treatment of atrial fibrillation (AF), including prediction models, enhanced electrocardiographs (AI-ECG), photoplethysmography, risk stratification, and intracardiac signal analysis. Highlighted successes, limitations, and future directions.	Various AI algorithms, including machine learning and deep learning	Provides insights into how AI can enhance AF detection and management, potentially improving screening and treatment strategies for athletes with AF.	Limitations include lack of racial diversity in training/testing cohorts, concerns about data integrity, potential misdiagnosis, operator dependence, and the need for further validation and calibration.
14	Bos JM, et al. (2021) [23]	Diagnostic case-control study	The AI-ECG model successfully distinguished patients with long QT syndrome from those without and provided a simple and inexpensive method for early detection of congenital long QT syndrome.	Deep neural networks, convolutional neural network	Although not directly mentioned, the ability to diagnose and manage cardiac conditions like LQTS is crucial in sports medicine for athlete safety.	Limited to a single-centre study without external validation; focused on a highly specific patient group (genetic heart rhythm clinic patients), which may limit generalizability.
15	Raissi Dehkordi N, et al. (2023) [24]	Review Article	AI enhances the accuracy and efficiency of ECG interpretation for diagnosing Long QT Syndrome (LQTS). AI algorithms reduce interobserver variability and can identify risk individuals by analyzing subtle ECG features not apparent to the human eye. AI-driven devices like smartwatches may facilitate early detection of LQTS-related complications.	Deep learning, neural networks	Provides tools for early detection and continuous monitoring of LQTS in athletes, potentially improving safety and preventive care in sports medicine.	Challenges include potential bias in AI training data, patient privacy concerns, and the need for enhanced interpretability of AI decisions to gain trust among clinicians.
16	Vozzi F, et al. (2022) [25]	Diagnostic Study	Developed a novel system using Echo State Networks (ESNs) for diagnosing type 1 Brugada Syndrome (BrS) with good accuracy. The system uses ECG pattern recognition, showing good performance particularly when using lead V2 only.	Recurrent Neural Networks (Echo State Networks)	Can potentially improve early detection of BrS in athletes, aiding in safer participation in sports by identifying individuals at risk of arrhythmias.	Larger datasets needed to improve model performance and validate its effectiveness in clinical practice.
17	Nakamura T, et al. (2023) [26]	Diagnostic Study	An AI model using a convolutional neural network predicted the presence of ventricular fibrillation (VF) in patients with Brugada syndrome from ECGs with high precision and reliability.	Convolutional Neural Network	Useful for predicting VF in athletes with Brugada syndrome, potentially preventing sudden cardiac death during sports activities.	Requires further validation in broader clinical settings to enhance reliability and determine general applicability.
18	Melo L, et al. (2023) [27]	Diagnostic Study	Developed a deep learning model to diagnose Brugada Syndrome (BrS) from ECGs without the need for sodium channel blockers. The model achieved high accuracy and AUC, indicating potential for clinical application to detect BrS more safely and effectively.	Deep Neural Network	Enhances the detection of BrS in athletes, potentially allowing for earlier and safer identification of those at risk of sudden cardiac death.	Further validation required to confirm effectiveness and generalizability in diverse clinical settings.
19	Zanchi B, et al. (2023) [28]	Diagnostic Study	AI model can identify Brugada syndrome based only on P-wave characteristics from ECGs, showing an alternative diagnostic pathway beyond the typical ventricular phenotype analysis. This underscores an atrial aspect to BrS pathology with practical applications in diagnosis using AI.	Machine Learning (various classifiers, including AdaBoost)	Could aid in identifying at-risk athletes with atrial abnormalities potentially linked to BrS, enhancing preventive measures in sports contexts.	Larger and more diverse datasets are required to enhance the robustness and applicability of the model in clinical settings.
20	Kwon J myoung, et al. (2021) [29]	Diagnostic Study	Developed a deep learning model (DLM) to detect various electrolyte imbalances (e.g., hyperkalemia, hypokalemia, hypernatremia, hyponatremia, hypercalcemia, hypocalcemia) using ECG data. Achieved high AUCs in both internal and external validations, demonstrating effectiveness in non-invasively detecting these conditions.	Deep Learning Model (DLM)	Could significantly aid in monitoring electrolyte status in athletes, particularly for those undergoing intense training or endurance sports, where imbalance risks are elevated.	Further validation with prospective studies and in different clinical environments is needed to generalize the findings.
21	Aro AL, Jaakkola I (2021) [30]	Editorial	AI applications in cardiology, especially in ECG interpretation, show high efficacy in diagnosing conditions like left ventricular ejection fraction (LVEF) ≤ 35% and hypertrophic cardiomyopathy (HCM). Attia et al. reported AI-ECG algorithms from the Mayo Clinic demonstrating high discrimination abilities with impressive AUC, sensitivity, and specificity.	Deep Learning Algorithms	AI-ECG could significantly enhance screening for heart conditions in athletes, potentially identifying risks early.	Editorial does not discuss specific limitations, but general challenges include data diversity and algorithm transparency.
22	Siontis KC, et al. (2023) [31]	Research Article	A convolutional neural network (CNN) model using 12-lead ECG and one-lead median-beat saliency maps demonstrated effective identification of hypertrophic cardiomyopathy (HCM). The study highlighted specific ECG segments, such as the ST-T segment, atrial depolarization, and QRS complex, which were crucial for the CNN model’s performance in detecting HCM. The use of saliency maps helped reveal which segments of the ECG were most influential in diagnosing HCM.	Convolutional Neural Network (CNN)	AI-based ECG analysis could be crucial for early detection of HCM in athletes, allowing for timely intervention and management to prevent severe cardiac outcomes.	The study was limited by the subjective interpretation of saliency maps and only included patients with a high AI-ECG likelihood of HCM, potentially biasing the results towards already known cases of HCM.
23	Adetiba E, et al. (2017) [32]	Research Article	Developed an automated heart defect detection model using ECG and Artificial Neural Network (ANN) for athletes. The model achieved classification accuracy, sensitivity, and specificity of 90.00%, 91.96%, and 97.06%, respectively, and identified four classes of heart conditions. The model can help reduce sudden cardiac death among athletes by enabling early detection of heart defects.	Artificial Neural Network (ANN)	Implementing this model in sports could help monitor athletes’ heart health in real-time, potentially preventing sudden cardiac death.	Limited discussion on the real-world applicability and integration into daily athletic monitoring. Lack of validation in diverse athletic populations.
24	Lyon A, et al. (2018) [33]	Research Article	Used mathematical modeling and machine learning to analyze high-fidelity 12-lead Holter ECGs from HCM patients, identifying distinct phenotypic subgroups. The study linked these subgroups with variations in clinical risk factors and anatomical features, providing insights that might help refine risk stratification for sudden cardiac death in HCM patients.	Machine Learning, Mathematical Modeling	Advanced ECG phenotyping may refine risk stratification and aid in early diagnosis of conditions leading to sudden cardiac death in athletes, potentially improving management and preventive strategies.	The study’s scope and impact on practical clinical outcomes were limited by the size and specific characteristics of the patient population studied.
25	Ko WY, et al. (20220) [34]	Research Article	This study developed an AI approach using a convolutional neural network (CNN) to detect hypertrophic cardiomyopathy (HCM) based on 12-lead ECGs. The CNN was trained on a large dataset and achieved high diagnostic performance, particularly in younger patients, suggesting potential for broad HCM screening applications.	Convolutional Neural Network (CNN)	The AI-ECG model’s high accuracy in detecting HCM could revolutionize cardiac health monitoring in sports, offering a non-invasive and efficient screening tool for early detection.	Requires further refinement and external validation to ensure its effectiveness across diverse populations and clinical settings.
26	Orini M, et al. (2023) [35]	Research Article	Investigated the association between premature ventricular and atrial contractions (PVCs and PACs) detected on wearable-format ECGs and cardiovascular outcomes in individuals without cardiovascular disease (CVD). Found strong associations between PVCs and heart failure (HF) and between PACs and atrial fibrillation (AF), suggesting that wearable ECGs could be useful for early cardiovascular risk stratification.	None Specified	Wearable ECG monitoring of premature contractions could enhance early detection and management of cardiac risks in athletes, potentially improving preventative care and outcomes.	Study findings are based on associations and require further investigation to confirm causality and practical applications in routine clinical practice.
27	Inder M, Diwakar IB (2023) [36]	Initiative Description Blog	Developed an AI model to interpret pediatric ECGs for SCA risk, achieving high sensitivity and specificity.	Machine Learning, Deep Learning	Direct application in preventing sudden cardiac arrests in athletes through early detection.	Requires further validation in clinical settings; data on long-term outcomes are lacking.
28	Derman W, et al. (2019) [37]	Review Article	Outlines the potential of AI in improving sports performance through enhanced heart rate monitoring and analysis.	Artificial Neural Networks, Support Vector Regression	Can inform training regimes and monitor health in real-time, enhancing athlete performance and safety.	More empirical data needed to substantiate the theoretical claims; commercial accessibility and integration issues.
30	Perez MV, et al. (2019) [38]	Prospective Study	Smartwatch application effectively identified atrial fibrillation with high predictive value; a low notification rate may indicate specificity.	Smartwatch algorithm, Irregular pulse notification	Potential for early detection of atrial conditions in athletes, improving prevention strategies.	Limited follow-up response rate for ECG patch returns, generalizability concerns due to high attrition.

## Abbreviations

SCD, sudden cardiac death; ECG, 12-lead electrocardiogram; PPE, pre-participation examination; AI, artificial intelligence; HCM, hypertrophic cardiomyopathy; IOC, International Olympic Committee; EFSMA, European Federation of Sports Medicine Associations; ESC, European Society of Cardiology; ML, machine learning; DL, deep learning; ANNs, artificial neural networks; CNNs, convolutional neural networks; AUC, area under the curve; AI-ECG, artificial intelligence-enhanced 12-lead electrocardiogram; AF, atrial fibrillation; LQTS, long QT syndrome; BrS, Brugada syndrome; PVCs, premature ventricular contractions.
